# Slow Escape from a Helical Misfolded State of the Pore-Forming Toxin Cytolysin A

**DOI:** 10.1021/jacsau.1c00175

**Published:** 2021-07-13

**Authors:** Fabian Dingfelder, Iuri Macocco, Stephan Benke, Daniel Nettels, Pietro Faccioli, Benjamin Schuler

**Affiliations:** † Department of Biochemistry, University of Zurich, Winterthurerstrasse 190, 8057 Zurich, Switzerland; ‡ Department of Physics, Trento University, Via Sommarive 14, 38123 Povo (Trento), Italy; § SISSA, Via Bonomea 265, 34136 Trieste, Italy; ∥ INFN-TIFPA, Via Sommarive 14, 38123 Povo (Trento), Italy; ⊥ Department of Physics, University of Zurich, Winterthurerstrasse 190, 8057 Zurich, Switzerland

**Keywords:** protein folding, single-molecule spectroscopy, microfluidic mixing, molecular dynamics simulations

## Abstract

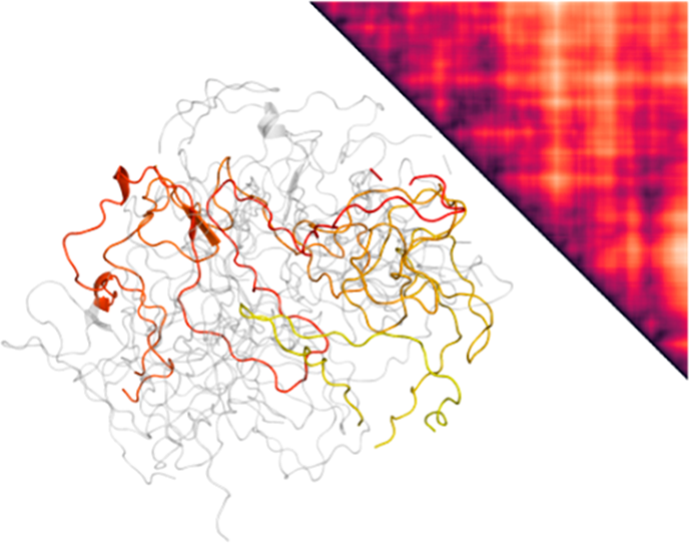

The pore-forming toxin cytolysin A (ClyA) is expressed as a large α-helical monomer that, upon interaction with membranes, undergoes a major conformational rearrangement into the protomer conformation, which then assembles into a cytolytic pore. Here, we investigate the folding kinetics of the ClyA monomer with single-molecule Förster resonance energy transfer spectroscopy in combination with microfluidic mixing, stopped-flow circular dichroism experiments, and molecular simulations. The complex folding process occurs over a broad range of time scales, from hundreds of nanoseconds to minutes. The very slow formation of the native state occurs from a rapidly formed and highly collapsed intermediate with large helical content and nonnative topology. Molecular dynamics simulations suggest pronounced non-native interactions as the origin of the slow escape from this deep trap in the free-energy surface, and a variational enhanced path-sampling approach enables a glimpse of the folding process that is supported by the experimental data.

Much of our current mechanistic understanding of protein folding is based on experiments with small single-domain proteins, which often fold on a millisecond time scale or faster, and whose kinetics can frequently be approximated with a two-state mechanism.^7,8^ Progress in the field over the past decade has greatly benefitted from the convergence of accessible time scales in experiments and simulations. On the one hand, experimental techniques are now able to monitor folding kinetics on shorter and shorter time scales, down to the folding speed limit in the microsecond range;^11^ on the other hand, simulations have extended their reach to longer and longer time scales, into the microsecond and millisecond range even for all-atom molecular dynamics.^12−15^ In contrast, the detailed folding mechanisms of larger proteins are still challenging to investigate, since the relevant time scales can extend to seconds, minutes, or even longer, often owing to the population of partially folded intermediates,^16^ which can be prone to misfolding and aggregation.^17,18^ Consequently, many large or multimeric proteins do not refold reversibly after dilution from denaturant solutions, and kinetically trapped states and competition with irreversible aggregate formation lead to complex kinetics and complicate quantitative analysis.^19−21^ However, large proteins, including membrane proteins, account for the majority of the proteome,^7^ so understanding their folding process is of great importance.

From the experimental side, structural and kinetic heterogeneity of folding processes can often be resolved by single-molecule techniques. For example, single-molecule force experiments have provided insight into the misfolding pathway of large biomolecular complexes such as Hsp90^22^ and have resolved misfolding events in single prion proteins.^17^ Single-molecule Förster resonance energy transfer (FRET) can be used to investigate time scales from nanoseconds to hours^23,24^ and has helped to elucidate, e.g., interdomain misfolding in tandem repeat proteins^25,26^ and complex folding kinetics.^27,28^ Furthermore, single-molecule FRET is especially well suited for investigating the dimensions and dynamics of non-native states.^29−32^ Here, we take advantage of the combination of single-molecule FRET with microfluidic mixing,^33^ a versatile tool for the investigation of nonequilibrium dynamics from milliseconds to minutes.^29,34^ We complement the distance information from FRET with circular dichroism (CD) spectroscopy to obtain equilibrium and kinetic information on secondary structure formation during folding.

A promising approach for investigating the complex folding mechanisms of large proteins is the combination of experimental techniques with simulations.^35,36^ While experimental techniques monitor global properties of the folding kinetics (such as the evolution of the average content of secondary structure) or probe the dynamics of a subset of degrees of freedom (such as specific inter-residue distances), computer simulations can in principle complement this information with a complete network of transient states that occur in protein folding^37^ or in receptor–ligand interactions,^38^ at an atomic level of resolution. This knowledge can even be exploited for pharmacological purposes, by identifying small molecules that stabilize on-pathway folding intermediates and trigger protein degradation.^39^ However, to be able to cover the relevant time scales of even moderately slow folding proteins in the range of seconds, additional approximations, such as coarse-graining, enhanced sampling techniques, or artificial biasing forces for accelerating the simulations must be introduced.^15,40−43^ Since such schemes may lead to systematic errors, it is particularly important to assess their reliability by direct comparison with experiments.

In this work, we studied the folding kinetics of the pore-forming toxin ClyA (34 kDa, 303 residues) from *E. coli* with a combination of single-molecule FRET, CD spectroscopy, and simulations using molecular dynamics (MD), and a recently developed variational enhanced path sampling technique, the bias functional approach.^44^ The large α-helical-bundle protein^4^ ClyA undergoes a large transition from the monomer to the protomer conformation upon interaction with membranes or detergent, which involves a rearrangement of 55% of its residues.^45^ The resulting protomer is then capable of assembling into dodecameric pores that penetrate the membrane and lead to the lysis of target cells.^46,47^ ClyA thus belongs to the group of bistable—or metamorphic^48^—proteins, which can assume two different stable folds. Here we investigate the folding of the ClyA monomer in the absence of membranes or detergent. Our experimental results reveal broadly distributed complex kinetics, ranging from a submillisecond collapse to the remarkably slow folding to the native state on the time scale of several minutes. These results also enabled us to assess the accuracy of a combination of unbiased and biased all-atom molecular simulations, which yielded an atomistic characterization of the folding process.

## Results

### Slow Folding of ClyA

**Figure 1:**
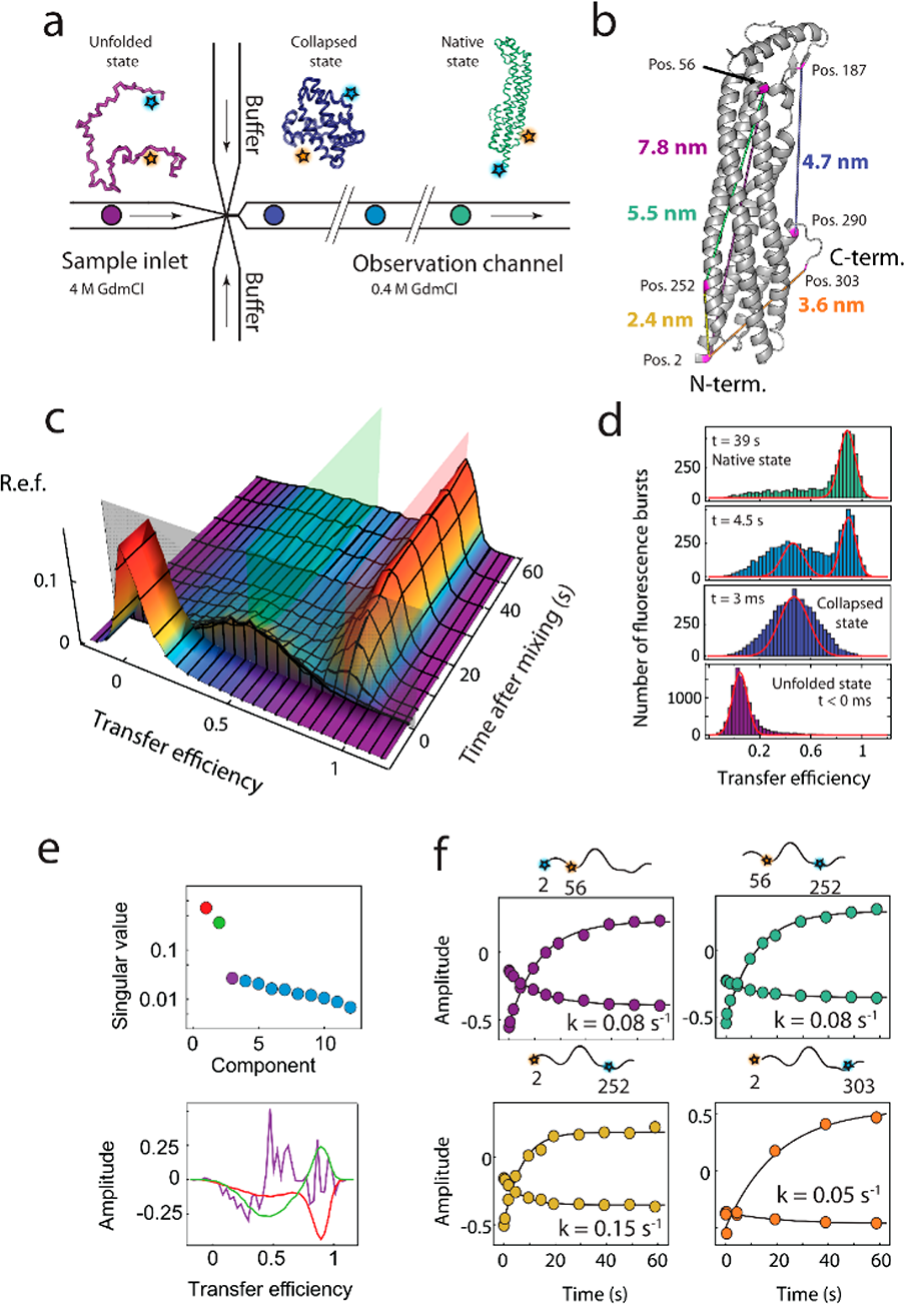
Folding kinetics of ClyA monitored by microfluidic mixing and single-molecule FRET. (a) Illustration of microfluidic mixing and single-molecule FRET along the observation channel. Upon rapid dilution of denatured ClyA (4 M GdmCl) with buffer solution, the protein rapidly collapses, followed by slow refolding to the native state. (b) Crystal structure of the ClyA monomer^4^ (PDB 1QOY) with the labeling positions and approximate interdye distances of the different variants indicated. (c) 3D plot of transfer efficiency histograms of ClyA 2C/252C as a function of time after mixing (unfolded state histogram prior to mixing). The time of mixing is indicated by the gray transparent plane. The mean transfer efficiencies of the collapsed and the native states are indicated as green and red transparent planes, respectively. R.e.f.: relative event frequency. (d) Individual transfer efficiency histograms of the data shown in (c) with the times after mixing indicated in each panel. Shot noise-limited peak shapes are indicated by red lines to illustrate the pronounced peak broadening in the collapsed state. (e) Singular value decomposition (SVD) of the transfer efficiency histograms after mixing (c) reveals two dominant singular values (top), with corresponding basis vectors in red, green and purple, respectively, illustrating that the third basis vector is dominated by noise (bottom). (f) Global fit of the time dependence of the two dominant components from singular value decomposition for four different ClyA variants with labeling positions indicated above the panels.

The folding kinetics of ClyA were investigated using confocal single-molecule FRET spectroscopy combined with a continuous-flow microfluidic mixing device with millisecond time resolution fabricated by replica molding with polydimethylsiloxane (PDMS) on a silicon mold.^33^ The protein was labeled with a donor- and an acceptor-fluorophore (Alexa Fluor 488/Alexa Fluor 594) at specific positions to generate different variants reporting on different segments of the polypeptide chain (Figure 1a,b and Figure S1). Protein unfolded in 4 M GdmCl supplied from the center inlet of the microfluidic device was rapidly diluted 10-fold by mixing with buffer solution supplied from the side inlets, which triggered the refolding process (Figure 1a). The resulting conformational changes as a function of time were monitored by placing the confocal observation volume at different positions along the observation channel, corresponding to different times after mixing (Figure 1c).^49^ For the variant T2C/E252C, for instance, the transfer efficiency histogram recorded in the sample inlet before mixing exhibits a peak at low transfer efficiencies (⟨*E*⟩ = 0.02), as expected for the expanded unfolded chain in denaturant^50−52^ (Figure 1c,d). Upon dilution of the denaturant, the polypeptide chain collapses within the 3 ms dead time of the measurements into a more compact state with ⟨*E*⟩ = 0.46. On the time scale of seconds, another population emerges and plateaus at about 1 min, with the transfer efficiency expected for the folded monomeric protein (Figure S1)^53^ (⟨*E*⟩ = 0.87, Figure 1d).

For a more quantitative kinetic characterization, the transition from the intermediate to the native state was analyzed from detailed time series of transfer efficiency histograms recorded in the microfluidic device for the different labeling variants (Figure 1c and Figure S2). Analysis of the refolding kinetics using singular value decomposition (SVD)^54,55^ revealed two components clearly separated from the noise (Figure 1e, top panel). For each protein variant, the first two components were well described by a global fit with single exponential functions sharing the same relaxation time. The different labeling variants yielded similar refolding rate coefficients, with *k* = 0.09 ± 0.04 s^–1^ (Figure 1f), suggesting that the conformational rearrangements on the tens of seconds time scale are a global process involving regions across the entire protein. The mean transfer efficiencies of the different variants in the native state reached after ∼1 min compare well to the values calculated based on the crystal structure (Figure S1), for which we take into account the accessible dye volumes^56^ and the interdye distance dynamics.^2,53^ One of the variants (A187C/K290C) could not be refolded in the microfluidic device but only by manual mixing (which does not allow kinetics faster than ∼1 min to be resolved, Figure S2). A potential reason might be that in this variant one of the fluorophores is positioned in the β-tongue of the native protein (Figure 1b), a hydrophobic β-hairpin that is thought to form the first contact upon interaction with membranes^57^ and could be a sensitive part of the protein whose destabilization may enhance interactions with hydrophobic surfaces such as the PDMS of the mixing device. In summary, microfluidic single-molecule FRET experiments reveal at least two steps during refolding of ClyA, involving first an initial rapid sub-3 ms collapse of denatured ClyA to an intermediate state, from which a state with native-like intramolecular distances emerges on a much longer time scale of tens of seconds. Identifying the structural and dynamic properties of the intermediate is thus likely to be key for understanding why the escape of ClyA to the native state is so slow.

### The Folding Intermediate of ClyA Is Compact Yet Highly Helical

**Figure 2:**
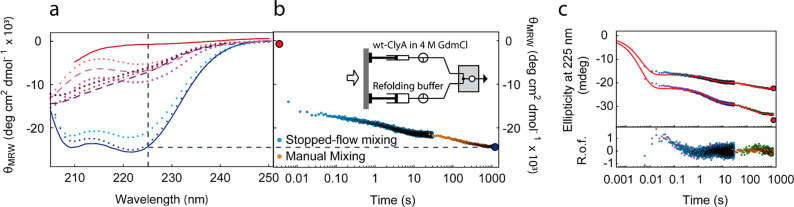
Circular dichroism measurements reveal helicity of the collapsed intermediate state. (a) Experimental and MD-derived equilibrium far-UV CD spectra of native and denatured ClyA, and CD spectra of the collapsed state calculated from MD simulations. Solid lines represent experimental data; all other spectra are derived from MD simulations (dashed lines: explicit-solvent simulations, obtained with the CHARMM36 force field, averaging over 20 configurations; dotted lines: implicit solvent simulations, obtained using the Amber 14SB force field, averaging over 15 configurations, except in the native state, where we used the energy-minimized structure. Red represents the unfolded state, blue the folded state, purple the collapsed intermediate. Two different servers were used to compute the spectra; light colors: PDB2CD,^9^ dark colors: PDBMD2CD(10)). The vertical dashed line indicates the wavelength at which kinetic refolding experiments were carried out (225 nm). (b) Refolding kinetics of 33 μM ClyA obtained by a combination of stopped-flow and manual mixing upon 11-fold dilution from 4 M GdmCl (inset). The red and blue points represent the equilibrium mean residue molar ellipticity of wt ClyA in 4 and 0.36 M GdmCl, respectively. (c) The same data as shown in (b) without normalization (top curve). Additionally, the experiment was repeated with higher initial concentration of ClyA (50 μM) (bottom curve). Describing the data requires at least three exponential components; a global fit of the two data sets with a triple-exponential decay and shared rate coefficients yields *k*_1_ = 235.2 s^–1^, *k*_2_ = 0.35 s^–1^, *k*_3_ = 0.007 s^–1^ (R.o.f.: residuals of the fit).

Kinetic traps in protein folding commonly involve the formation of transient non-native interactions and structure whose unfolding becomes rate-limiting.^40,58^ Often, such interactions involve β-structure, which can lead to persistent non-native hydrogen bonding within the polypeptide chain. To address the question of whether such interactions might be present in the intermediate we observe, we probe the secondary structure content in equilibrium (Figure 2a) and stopped-flow (Figure 2b,c) circular dichroism (CD) experiments where denatured wt-ClyA is refolded by rapid dilution. As depicted in Figure 2b, upon dilution of the denaturant, a large burst phase with ∼57% of the total amplitude in the ellipticity at 225 nm was observed within the 6 ms dead time of the measurement, indicating that a large amount of α-helical structure is formed already in the collapsed intermediate observed by single-molecule FRET. The dominant kinetic phase of structure formation that follows occurs with a rate of ∼0.35 s^–1^ if described as single-exponential, in a similar range as the dominant time scale observed in the single-molecule kinetics (Figure 1), but a deviation from single-exponential behavior is detectable at short times. Stopped-flow experiments carried out at different initial ClyA concentrations could be fitted with the same rate coefficients, indicating that protein aggregation does not have a pronounced effect on the observed folding kinetics (Figure 2c). However, the ellipticity after the 30-s acquisition time of the stopped-flow experiments does not reach the value of fully folded ClyA. Extended kinetics performed by manual refolding under the same solution conditions revealed an additional slow component on the minutes time scale (*k*_3_ = 0.007 s^–1^) (Figure 2b,c). To see whether the slow increase in helicity observed by CD spectroscopy is accompanied by intramolecular distance changes, manual-mixing single-molecule FRET measurements were also performed (Figure S2). However, no significant changes in the transfer efficiency histograms were detectable on a time scale above 1 min (Figure S2), indicating that the formation of the final 11% helicity does not have a detectable effect on the intramolecular distances probed by FRET and thus suggesting that the overall structure is reached within about a minute. Altogether, the broad range of time scales observed for secondary structure formation and the discrepancies with the kinetics observed by single-molecule FRET illustrate the complexity of the folding process after the rapid initial collapse.

**Figure 3:**
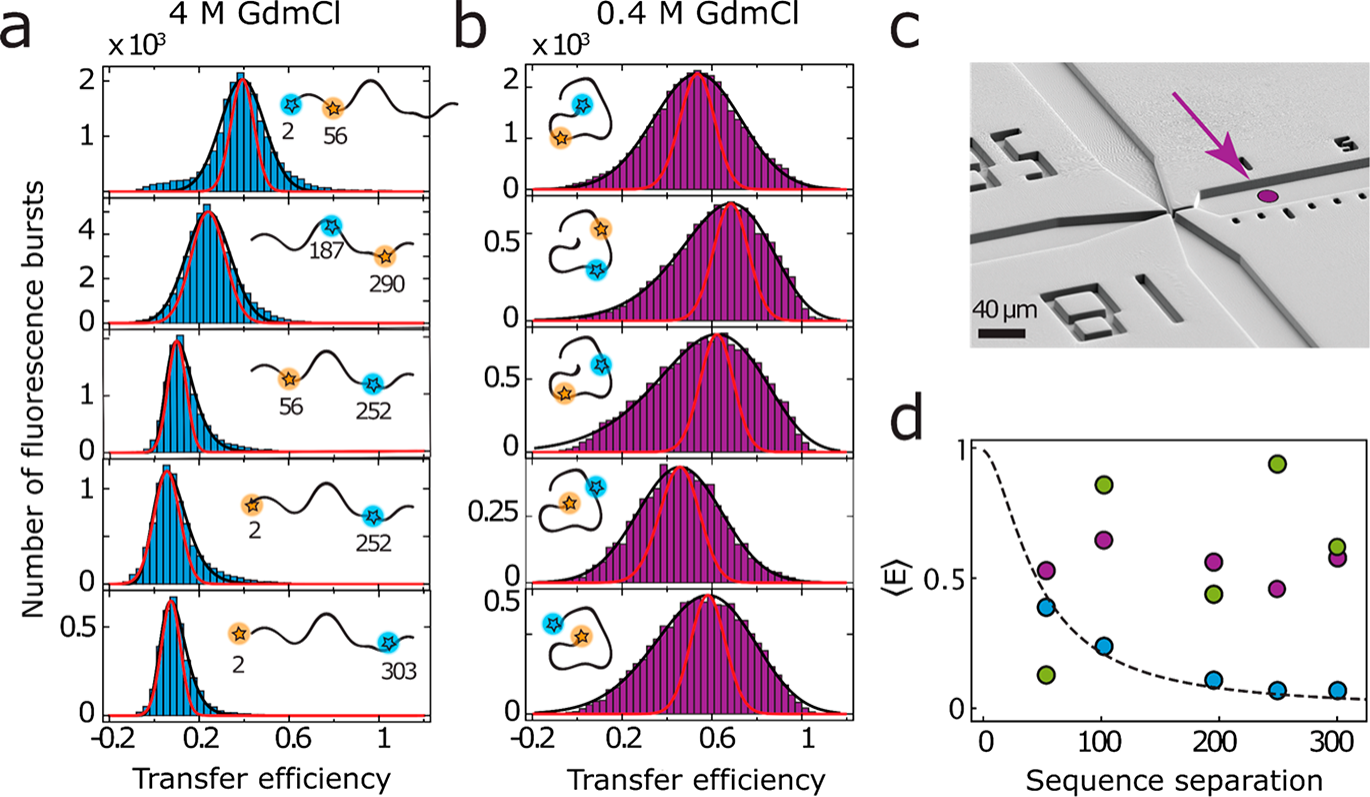
Mapping the collapsed intermediate of ClyA with single-molecule FRET. (a) Transfer efficiency histograms of the ClyA variants unfolded in 4 M GdmCl, with increasing sequence separation between the fluorescent dyes. (b) Transfer efficiency histograms of the collapsed intermediate state in 0.4 M GdmCl, recorded 40 ms after mixing the unfolded protein with buffer. Red lines show shot noise-limited peaks to illustrate the pronounced excess width of the histograms in the collapsed state. (c) Electron micrograph of the microfluidic device with the position of data acquisition highlighted by the magenta arrow. (d) Mean transfer efficiency of ClyA unfolded in 4 M GdmCl (blue), collapsed (magenta) and native (green) as a function of sequence separation. The dependence for the unfolded state is a fit using a self-avoiding walk model^6^ (dashed black line).

To assess the dimensions of the unfolded and the collapsed and highly helical intermediate states, we further compared the transfer efficiencies in the two states as a function of sequence separation of the FRET dyes in the different variants of ClyA, ranging from ∼50 to ∼300 amino acid residues (Figure 3a, Figure S2). Equilibrium single-molecule FRET measurements in 4 M GdmCl show the monotonic decrease in transfer efficiency with increasing sequence separation expected for an unfolded protein (Figure 3a,d). This behavior is well described quantitatively by a self-avoiding random walk model^6^ with a length scaling exponent of 0.6, reflecting the typical behavior of an expanded polypeptide chain unfolded in denaturant.^59,60^

To be able to probe the FRET efficiency of the kinetic intermediate as a function of sequence separation, it needs to be populated transiently. We thus compared the transfer efficiency histograms of the different ClyA labeling variants 40 ms after inducing the refolding process by rapidly diluting GdmCl-denatured ClyA with buffer in the microfluidic mixing device (Figure 3b,c). In contrast to the GdmCl-unfolded state, the mean transfer efficiencies in the intermediate do not decrease with sequence separation but rather fluctuate around a mean of ∼0.56 without detectable trend (Figure 3b,d). These results provide twofold evidence for a compact intermediate state: First, the average transfer efficiencies in the intermediate are higher than in the GdmCl-unfolded state. Second, the mean transfer efficiencies do not decrease with sequence separation, which is the behavior expected for a globule, i.e., a fully collapsed chain without well-defined long-range structure: in this case, the ensemble-averaged internal distances beyond the length scale of a few residues are independent of segment length and essentially determined by the monomer density within the globule.^61,62^ In such a compact and globule-like state, the fluorophores are thus on average in close proximity, even for large sequence separations between the dyes, resulting in a higher transfer efficiency compared to the GdmCl-unfolded state (see, e.g., the lowest panels in Figure 3a,b).^63^ The lack of correlation between the transfer efficiencies in the kinetic intermediate and in the native state further suggests a nonspecific collapse where the lengths and packing of helices observed in the folded state are not yet formed. The combined results from CD spectroscopy and single-molecule FRET thus indicate that the folding intermediate of ClyA is highly helical yet compact, in contrast to the elongated native helical bundle.

### Dynamics in the Intermediate

The compactness of the intermediate and the absence of native topology is suggestive of strong non-native interactions, which may be expected to lead to slow dynamics of reconfiguration within this collapsed ensemble. A first indication comes from the broad transfer efficiency histograms in the intermediate, whose width is in great excess of the value expected from shot noise (caused by the limited number of photons per fluorescence burst, Figure 3b). This broadening indicates large conformational heterogeneity with dynamics on a time scale greater than ∼1 ms,^64−66^ the molecules’ translational diffusion time through the confocal volume. Fluorescence anisotropies in the intermediate are between 0.10 and 0.23 (Figure S3), making restricted rotational donor and acceptor mobility unlikely to be the dominant cause of broadening in the transfer efficiency histograms.^67^

**Figure 4:**
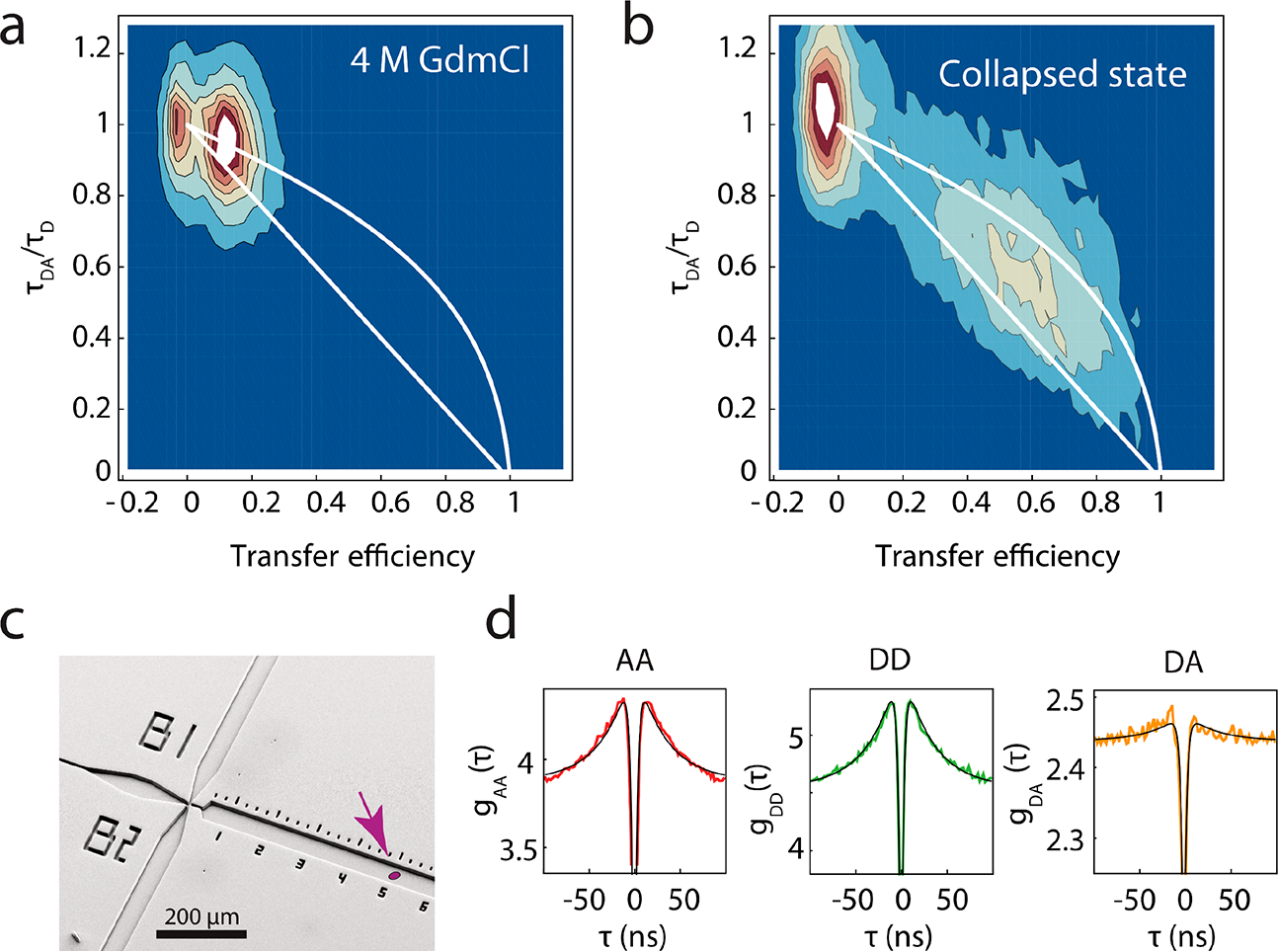
Slow dynamics in the collapsed intermediate. (a, b) 2D histograms of relative donor lifetimes versus transfer efficiencies determined for each burst for (a) the GdmCl-unfolded state of ClyA 56/252 and (b) the collapsed state measured in the microfluidic device 40 ms after mixing. The donor lifetimes in the presence and absence of acceptor are denoted as *τ*_DA_ and *τ*_D_, respectively. The straight white line indicates the expected dependence for a static interdye distance.^1−3^ The curved line is the expected dependence for a Gaussian chain with rapid fluctuations of the interdye distance.^5^ (c) Scanning electron micrograph with the measurement position indicated. The corresponding time after mixing is 380 ms at which the intermediate state is populated almost exclusively (Figure S1c). (d) Nanosecond fluorescence correlation spectroscopy (nsFCS) of the collapsed intermediate of ClyA 2/252 recorded in the microfluidic device. The panels indicate the autocorrelation of acceptor fluorescence [*g*_AA_(*τ*)], donor fluorescence [*g*_DD_(*τ*)], and the cross-correlation of donor and acceptor fluorescence [*g*_DA_(*τ*)]. The sharp decrease in the correlation curves for lag times approaching zero is due to photon antibunching. The solid black lines show a global fit with shared time constants for antibunching (*τ*_ab_) and rotational diffusion (*τ*_rot_) (see the Methods), yielding *τ*_ab_ = 2.7 ns and *τ*_rot_ = 33 ns.

Further evidence for the presence of slow dynamics comes from the analysis of fluorescence lifetimes. In two-dimensional histograms of donor fluorescence lifetime versus transfer-efficiency (Figure 4a,b), populations lacking distance dynamics are expected to be centered close to the diagonal line.^1−3^ Conversely, if distance fluctuations occur on time scales between the donor fluorescence lifetime and the translational diffusion time through the focus, the corresponding population departs from the diagonal and is shifted toward higher relative donor fluorescence lifetimes,^2^ as for ClyA unfolded in 4 M GdmCl (Figure 4a). The collapsed intermediate state of ClyA is located in between these two limiting regimes (Figure 4b), indicating distance dynamics that are much slower than for an expanded unfolded protein^68^ but also not fully static on the 1 ms time scale. The pronounced difference between fully unfolded ClyA and the collapsed intermediate is also apparent in nanosecond fluorescence correlation spectroscopy (ns-FCS)^69^ experiments in the microfluidic device (Figure 4c,d). For the intermediate, we find no indication for chain reconfiguration dynamics, since the donor–acceptor cross-correlation shows no anticorrelated signal on a 10–100 ns time scale that would be expected in case of rapid distance fluctuations.^29,69^ Instead, a component with a positive amplitude is observed not only in the donor–donor and acceptor-acceptor correlation functions but also in the donor–acceptor cross-correlation. Since direct excitation of the acceptor leads to a correlation decaying on the same time scale (Figure S4), the signal is most likely due to rotational diffusion of the entire protein.^70^ However, a contribution from static acceptor quenching cannot be excluded, although this effect typically occurs on a longer time scale.^71,72^

The combined structural and dynamic experimental data point toward an unusual type of folding intermediate in which a lot of helical structure is already formed but the helices are not of the lengths and in the packing arrangement of the folded structure. Rather, they form a compact globule-like state with slow dynamics indicative of pronounced non-native interactions that slow down the search for the native structure.

### Simulating Escape from a Compact Intermediate

**Figure 5:**
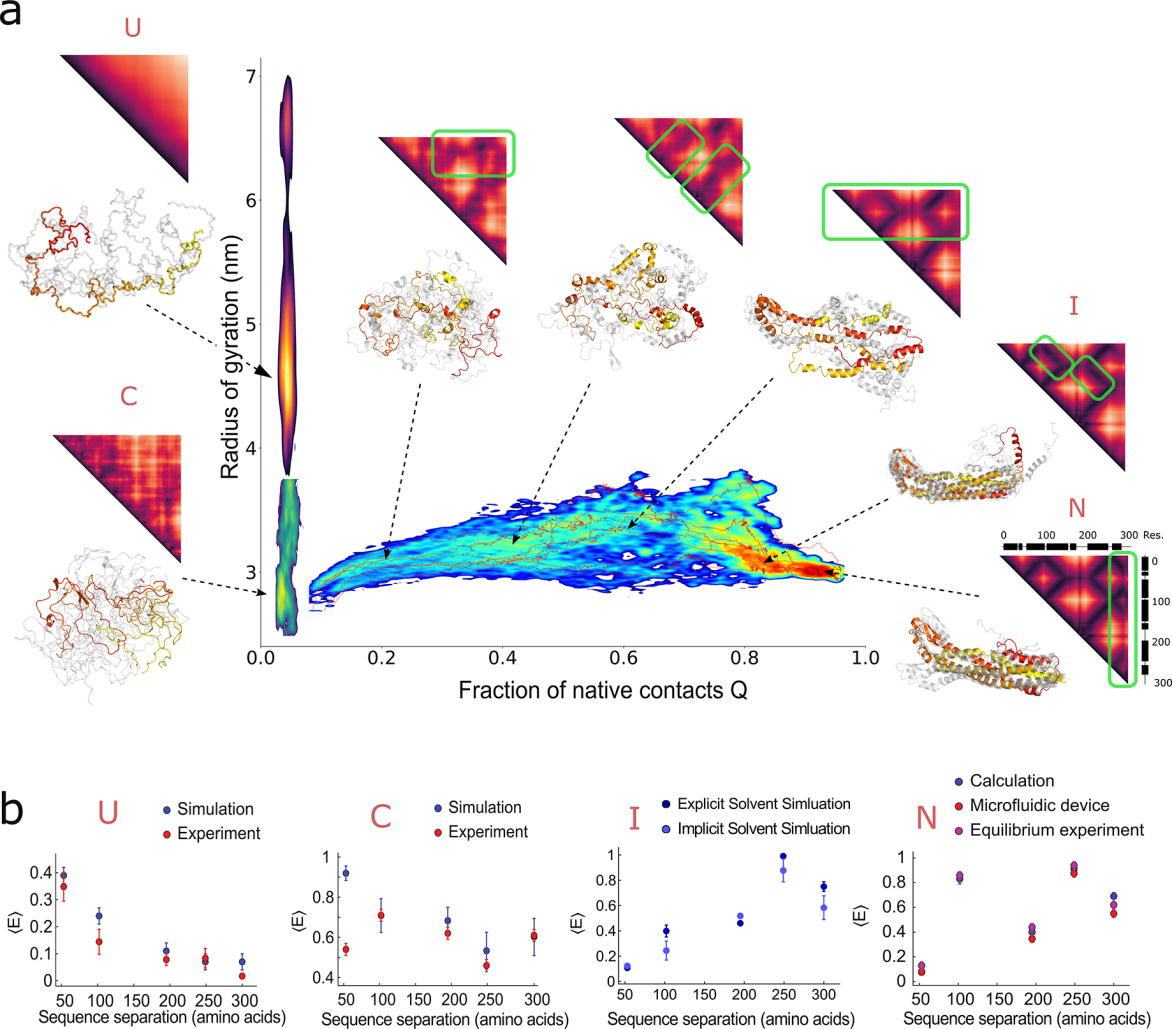
Summary of the refolding mechanism obtained with MD simulations and comparison with experimental results. (a) Density plots showing the unfolded high-temperature ensemble (“U”), the collapsed intermediate (“C”), and a set of folding trajectories obtained by a bias functional method projected onto the plane selected by the fraction of native contacts *Q* and the radius of gyration. All these configurations were extracted from simulations performed in explicit solvent (CHARMM36). The bias functional folding trajectories were started from initial conditions in C that were obtained by independent short (1.5 ns) implicit solvent MD starting from U. This procedure was adopted to ensure the statistical independence of the initial conditions. The heat map extending from low to high *Q* denotes the frequency histogram calculated from the entire ensemble of ratchet-and-pawl MD trajectories, and the thin red lines are the least-biased trajectories selected by the variational criterion (see Methods). Representative structural ensembles and intramolecular contact maps are shown for different stages of the folding process (black indicates high and yellow low contact probability; green frames indicate the regions in the contact map where new native interactions are formed; the helical stretches along the sequence are indicated schematically for the native state contact plot). The structural ensembles show overlays of five configurations with one highlighted in color. “I” denotes a near-native folding intermediate observed in the simulations and “N” the native state. (b) Comparison of experimental mean transfer efficiencies with results from MD simulations for the four different states indicated in (a).

In the next step, we aim to obtain a more detailed structural picture of the intermediate and the escape to the native state from molecular simulations. The starting point for ClyA folding in the experiments is the fully unfolded state at high denaturant concentration. To model this highly expanded ensemble, we generated coil-like chain structures in high-temperature (800 K) plain MD simulations using Amber 14SB with implicit solvent (Figure 5a) (see the Methods). The average FRET efficiencies calculated from this ensemble for the different donor–acceptor pairs are close to the experimental results (Figure 5b), and the helicity is low (Table S1), consistent with the results from CD spectroscopy in 4 M GdmCl (Figure 2a), suggesting a suitable representation. Upon dilution of the denaturant, the ensemble of expanded coil-like configurations is very far from equilibrium and will undergo rapid collapse, observed as the burst phase in the dead time of the experiments. To model this process, we performed five independent 500 ns MD simulations in explicit solvent (four with CHARMM36^73^ and one with Amber14SB^74^) and 15 1.5 ns simulations in implicit solvent with the Amber14SB force field, starting from fully unfolded configurations. We used these trajectories to compute the time dependence of the radius of gyration, *R*_g_, the fraction of native contacts, *Q*, the helicity, *h*, relative to the native structure, and the distances between the pairs of residues monitored in the FRET experiments. These collective variables (CVs) change in a way consistent with very rapid collapse to a globular ensemble (Figure S5). In the explicit-solvent simulations, this collapse occurs on a time scale of 100–300 ns, close to typical reconfiguration time of unfolded proteins (∼20–200 ns),^5,68^ which, according to Onsager’s regression hypothesis, is expected to be similar to the collapse time.^75^

To quantify the structure of the collapsed state in the simulations, we randomly sampled configurations from the plateau region of the MD trajectories. The average values of *R*_g_, *Q*, and *h* are reported in Table S1, FRET efficiencies in Figure 5. These results show some of the key properties of the kinetic ClyA intermediate identified experimentally. In particular, the calculated FRET efficiencies are close to the experimental values for four of the five distance pairs (Figure 5b). Only for variant 2C/56C, we find a clear discrepancy, possibly indicating that the lengths of individual helices are not equilibrated in our simulations. To investigate this possibility, we (i) directly computed the helicity with the DSSP algorithm^76^ and (ii) estimated CD spectra using the PDB2CD^9^ and the PDBMD2CD servers.^10^ The CD spectra computed from ensembles of configurations in the molten globule and unfolded states vary significantly depending on the server used (see Figure 2a). We thus also estimated the relative helicity using the DSSP algorithm,^76^ resulting in average values ranging from ∼1% to slightly over 20%, depending on the force field (see Table S1 and Figure S5). Despite this variability, both simulations and experiment exhibit an increase in helicity from the fully unfolded to the collapsed state, but the experimental increase is more pronounced (Figure 2). The remaining discrepancy may thus indeed be caused by incomplete local equilibration of helix formation in the simulations in the collapsed state.

The fraction of native contacts in the ensemble of configurations in the collapsed state obtained from explicit-solvent MD simulations is less than 10% (Figure 5), but pronounced non-native interactions are present, which are expected to slow down the conformational relaxation in the collapsed intermediate and hinder the escape to the native structure. Altogether, the simulations thus capture important characteristics of the kinetic folding intermediate of ClyA as a collapsed globular ensemble that exhibits helical content but lacks the arrangement of helices in the native structure and shows slowed dynamics compared to a fully unfolded protein; it thus resembles a molten-globule-like configuration.^77^

The experimental data indicate that the slow transition from the molten-globule-like intermediate to the native state occurs on a time scale of seconds to minutes, out of reach for unbiased MD simulations. We thus employed an enhanced path sampling method based on a variational approximation, using the Amber 14SB force field.^74^ Specifically, we used ratchet-and-pawl MD^78,79^ (rMD) to efficiently generate a statistically significant number of folding trajectories. In rMD, an unphysical history-dependent force is introduced to prevent the chain from backtracking toward the reactant, along the direction defined by some suitably but arbitrarily chosen collective variable (CV). Conversely, the biasing force remains latent when the system spontaneously progresses toward the product. It has been shown that rMD samples the Boltzmann distribution in the transition region in the limit where the CV used is the committor function.^80^ In practical calculations, the chosen CV provides only a proxy of the ideal reaction coordinate. However, it is still possible to keep systematic errors to a minimum by applying the bias functional (BF) method.^44^ In this approach, a variational principle derived from Langevin dynamics is used for scoring the folding trajectories generated by rMD to identify those with the highest probability of occurring in the absence of any biasing force. The approach has been shown to identify reaction paths consistent with equilibrium MD simulations.^81^

Figure 5 illustrates examples of resulting trajectories sampling the transition from the intermediate to the folded state. The density map, obtained from a frequency histogram of the calculated rMD trajectories, provides a qualitative estimate of free energy landscape. The trajectories and the intramolecular contact maps suggest that the transition from the early collapsed intermediate to the folded state occurs within the compact ensemble, impeded by pronounced non-native interactions. A first major step comprises the formation and packing of helices αB, αC, and αF, which make up the structural core of the helix bundle. Interestingly, this core has also been identified as a stable equilibrium intermediate in denaturant-induced unfolding experiments,^53^ supporting the relevance of the simulation result. The simulations suggest the docking of helix αA to be the next step. The resulting native-like intermediate, with a fraction of native contacts >0.8 and a helical content close to that of the native state, is rather long-lived, an observation that may be related to the slowest phase of structure formation in the CD experiments. The FRET efficiencies in this state (Figure 5b, I) show that four out of five residue pairs are in a native-like configuration. Variant 187/290 would be expected to probe the existence of this intermediate most directly, but since it fails to refold in the microfluidic device, it does not provide any information on this question. The simulations suggest that the solvent-accessible surface area of hydrophobic residues in this intermediate (I) is about 40% greater than in the native state, which may lead to stronger interactions with the surfaces of the microfluidic device and hamper refolding. The final folding step observed in the simulations is the slow docking of the most C-terminal helix αG, which leads to the final fully folded structure of ClyA.

## Discussion

We used single-molecule FRET and microfluidic mixing combined with CD spectroscopy to probe the folding dynamics of the bacterial toxin ClyA, a large helical protein of 303 residues that interacts with the membranes of target cells to form membrane-penetrating pores. We identified a kinetic folding intermediate that forms within the 3 ms dead time of mixing upon dilution of denaturant and that has interesting conformational and dynamic properties: (i) The mean transfer efficiencies of labeling pairs within this state are higher than in the denaturant-unfolded state and insensitive to the sequence separation of the fluorescent dyes, indicating a compact globule-like conformational ensemble. (ii) Stopped-flow CD measurements reveal high helix content in the intermediate, but (iii) the transfer efficiencies in the intermediate are uncorrelated with those in the native state, indicating non-native lengths and relative arrangements of the constituent helical segments. (iv) The pronounced broadening of transfer efficiency histograms and fluorescence lifetime analysis suggest that long-range dynamics within the intermediate are much slower than in more expanded unfolded proteins. (v) Conversion from the intermediate to the native state occurs very slowly, on the time scale of minutes.

Key properties of the intermediate are reproduced in all-atom explicit-solvent MD simulations: Starting from expanded random-coil configurations, the chain collapses rapidly (in ∼100 ns) to a compact ensemble, with good overall agreement of the calculated transfer efficiencies and average helicity with the experimental values. Similar to the experimental findings, the dynamics in the compact intermediate are slowed down relative to the expanded chain, suggesting considerable non-native interactions within this molten-globule-like state. A recently developed variational enhanced path sampling technique, the bias functional approach,^44^ enabled us to simulate the slow escape from the intermediate to the native state, which requires excursions to more expanded states with high free energy, from which folding to the native state is possible.

The combination of experiment and simulations thus provides novel insight into the folding process of a protein of such large size, which can be illustrated in terms of the free-energy surface approximated from the simulations (Figure 5a). Using the radius of gyration and the fraction of native contacts as reaction coordinates, the starting point is the highly expanded chain with the dimensions of the protein in 4 M GdmCl. Upon dilution of denaturant (or the jump to low temperature in the simulations), the chain collapses rapidly to a compact ensemble with high helicity but a low fraction of native contacts and large conformational heterogeneity. Owing to the non-native interactions and the pronounced compaction of the intermediate, reconfiguration within this ensemble is sluggish, and finding the correct length and arrangement of the long helices of ClyA is an accordingly slow process that requires rare large conformational fluctuations out of the molten-globule-like intermediate, which corresponds to a deep trap in the free-energy surface.

On the path to the native state, the simulations identify the possibility of an on-pathway intermediate, in which the C-terminal αG helix is not yet docked onto the helix bundle. Directly testing the presence of this intermediate in the microfluidic single-molecule experiments used here was not possible. However, previous single-molecule denaturation experiments indicated the existence of an equilibrium folding intermediate of ClyA at low GdmCl concentration in which the terminal helices are denatured, whereas the rest of the helix bundle remains intact, corresponding to relatively weak packing interactions of the terminal helices. The existence of the kinetic on-pathway folding intermediate observed in the simulations would thus be plausible.

Although detailed structural information on folding intermediates of proteins the size of ClyA is sparse, several proteins have been shown to exhibit related behavior. Walters et al. investigated the folding pathway of maltose binding protein, which is comparable in size to ClyA, by hydrogen exchange combined with mass spectrometry.^16^ Within the experimental dead time of a few milliseconds, the authors observed the formation of extensive helical content as well as a collapse into an ensemble of globule-like states. The subsequent conversion to the native state occurs on a similarly slow time scale as for ClyA. Membrane proteins have also been shown to exhibit behavior reminiscent of what we overserved here for ClyA. An example is the membrane-embedded rhomboid protease Glp, which showed almost identical far UV-CD spectra for the SDS-denatured state and after renaturation in mixed SDS/DDM micelles.^82^ Thus, also in this case, folding is dominated by the rearrangement and assembly of helical segments. Another interesting case is the slow interconversion and conformational heterogeneity reported for the unfolded state of the β-barrel outer-membrane protein phospholipase A detected by single-molecule spectroscopy.^32^

The behavior of these proteins contrasts with many small single-domain proteins, whose folding is often fast, well approximated by a two-state mechanism, and lacks the population of intermediates.^83^ In these cases, native and near-native interactions typically dominate the folding process, leading to smooth free-energy surfaces and efficient funneling toward the native state.^84,85^ Avoiding non-native interactions appears to be much more difficult for large proteins, where the larger number of possible non-native interactions is bound to favor compact ensembles that constitute efficient traps on the energy landscape and lead to the exceedingly slow folding often observed.^20^ Interestingly, such non-native interactions occur not only for proteins rich in β-structure, where promiscuous hydrogen bonding is suggestive of strong non-native interactions,^58^ but also for proteins like ClyA, whose secondary structure is dominated by α-helices, even in the intermediate. Many helical transmembrane proteins may thus face similar challenges in their folding process.^86,87^ The formation of stable intermediates and collapsed ensembles might be prevented in vivo by binding to molecular chaperones that keep the polypeptide chain in an expanded conformation,^88^ either on or off the ribosome;^89^ subsequent release can allow a transition to the native state, either directly, e.g., by stepwise release into the native membrane environment^86,87^ or in an iterative annealing mechanism.^90^

## Materials and Methods

### Protein Expression, Purification, and Labeling

We selected labeling positions in ClyA that are surface-exposed in both the monomer and the protomer conformation according to the available structural data.^4,46^ ClyA was expressed, purified, and labeled as described previously.^47^ Briefly, the double-cysteine variants (T2C/Q56C, A187C/K290C, Q56C/E252C, T2C/E252C, and T2C/V303C) were produced by site-directed mutagenesis of the pClyA vector coding for N-terminally His_6_-tagged ClyA, using the QuickChange protocol (Stratagene). Protein expression was carried out in *E. coli* Tuner DE3 (Merck Millipore) at 20 °C for 12 h. The double-cysteine variants as well as wt-ClyA were purified, via immobilized metal affinity chromatography (IMAC) with a nickel-nitrilotriacetic acid (NTA) resin (Thermo Fischer). ClyA-wt for the CD experiments was buffer exchanged to 10 mM sodium phosphate (pH 7.4) on a HiPrep 26/100 desalting column (GE Healthcare) and further purified by anion-exchange chromatography on a HighPrep QFF 16/100 (GE Healthcare) applying an elution gradient of 0–1 M NaCl.^47^ The protein was then denatured by the addition of GdmCl to a final concentration of ∼6 M and concentrated to 230 μM. For final purification, size exclusion chromatography on a Superdex 200 Increase 10/300 column (GE Healthcare) was performed, yielding a final protein concentration of 50 μM.

Following nickel chelate affinity chromatography, the double-cysteine variants for labeling were concentrated to ∼400 μM and reduced by the addition of dithiothreitol (DTT) to a final concentration of 10 mM. The reducing agent was removed by a HiTrap desalting column (GE Healthcare) using a buffer containing 50 mM sodium phosphate and 150 mM NaCl (pH 7.3). For donor labeling, Alexa Fluor 488 C5 maleimide (Invitrogen) dissolved in dimethyl sulfoxide (DMSO) was added in a dye-to-protein molar ratio of 0.7:1 The reaction was carried out at room temperature for 2 h and quenched by the addition of DDT to a final concentration of 50 mM. After removal of free dye and buffer exchange to 10 mM sodium phosphate (pH 7.3) via a HiTrap desalting column (GE Healthcare), the singly labeled protein was separated from unlabeled and doubly labeled protein by anion exchange chromatography, using a MonoQ GL column (GE Healthcare). The protein was concentrated to ∼100 μM and Alexa Fluor 594 C5 maleimide (Invitrogen), dissolved in DMSO, was added in 3-fold molar excess. The labeling reaction was carried out overnight on ice and then quenched by the addition of DDT to a final concentration of 50 mM. Unreacted dye was removed by a HiTrap desalting column, followed by anion-exchange chromatography. The mass of all variants was confirmed by electrospray ionization mass spectrometry. Variant 56/252 was previously tested for hemolysis activity to ensure functionality in pore formation.^47^ For the dye-labeled variants 2/56, 187/290, 56/252, and 2/252, conversion to the protomer conformation was verified in the presence of the membrane-mimicking detergent dodecyl maltoside via single-molecule FRET, which agreed with the transfer efficiencies expected for the protomer conformation.

### CD Spectroscopy

Equilibrium spectra (Figure 2) were recorded on a JASCO J715 CD spectrometer at 293 K. For equilibrium measurements of refolding to native ClyA, the 50 μM stock solution in 4 M GdmCl was diluted 11-fold with buffer (25 mM sodium phosphate, 75 mM NaCl, pH 7). The samples were incubated for at least 1 h, data were recorded in a 1 mm path length cuvette in the range of 200–250 nm with a scanning speed of 10 nm/min, and 15 spectra were averaged. For the measurement in 4 M GdmCl, a 10-μM sample was measured in a 0.5 mm path length cuvette, and 20 accumulations were averaged. The data are shown from 210 to 250 nm owing to pronounced absorption of the denaturant at lower wavelengths. For manual mixing experiments, 25 μL of the stock solution was rapidly diluted with 250 μL of buffer solution (dead time ∼15 s), and the ellipticity at 225 nm was recorded every 5 s for 20 min. To test the concentration-independence of refolding rate coefficients, manual mixing experiments were repeated with a 33 μM stock solution in 4 M GdmCl, with measurements in triplicate (Figure 2c).

Stopped-flow measurements were performed on a PiStar spectrometer (Applied Photophysics) with a 2 mm path length cell. Refolding was triggered by rapid 11-fold dilution of wt-ClyA denatured in 4 M GdmCl with buffer (25 mM sodium phosphate, 75 mM NaCl, pH 7). A total of 5000 data points were recorded over a period of 30 s, with the first point measured 6 ms after mixing. For the initial concentrations of 50 μM and 33 μM, eight and seven shots were averaged, respectively (Figure 2b,c). Some traces had to be discarded due to drift within the 30 s recording interval. For combining the kinetic traces obtained by manual mixing and stopped-flow measurements, the stopped-flow data were multiplied by 0.97 and 1.07 for 50 and 33 μM initial concentrations, respectively, to compensate for small differences in absolute values between the data sets. The combined data were globally fitted with a triple-exponential fit that was constrained to the ellipticity measured in 4 M GdmCl at time zero. The rate coefficients were shared fit parameters between the data sets and the amplitudes free fit parameters (Figure 2c). Stopped-flow experiments at the different initial ClyA concentrations of 33 and 50 μM could be well described with the same rate coefficients (Figure 2c), indicating that protein aggregation does not have a pronounced effect on the observed folding kinetics.

### Single-Molecule Spectroscopy

Single-molecule measurements were performed on a MicroTime 200 confocal instrument (PicoQuant) at 295 K, as described previously.^91^ Pulsed interleaved excitation^92^ (PIE) was applied to remove the contribution of molecules lacking an active acceptor dye. The emitted photons after donor- and acceptor excitation were collected via an UplanApo 60/1.2W objective (Olympus) and split first according to polarization (polarization cube, PicoQuant) and then according to color (595 DCXR, Chroma). Manual refolding experiments were carried out by rapidly diluting 5 μL of labeled protein (∼1.5 nM) with 45 μL of buffer solution (25 mM sodium phosphate, 75 mM NaCl, 0.001% Tween 20, pH 7), and transfer efficiency histograms were recorded for at least 20 min (dead time ∼20 s). For data analysis, the histograms were split into 2 min time intervals. To sample a sufficient number of molecules in each 2 min time window, the fluorescent bursts of 3–11 measurements were added.

### Microfluidic Mixing Experiments

The microfluidic mixing device covers time scales from milliseconds up to 1 min.^47,93^ Replica molding and interfacing with single-molecule instrumentation was performed as described previously.^33^ To reduce nonspecific surface interactions of the sample molecules with the channel walls, the microfluidic devices were passivated prior to single-molecule measurements. The ClyA variants T2C/Q56C, A187C/K290C, Q56C/E252C, and T2C/V303C were passivated by flushing the microchannels with unlabeled wt-ClyA in 4 M GdmCl for 1 h. Subsequently, the device was flushed with 4 M GdmCl for at least 20 min. For ClyA T2C/E252C, the block copolymer Pluronic F127 (200 mg/mL in ethanol) was added for surface passivation to the PDMS precursors (2 μL/g PDMS) prior to curing.^94^ The 25 × 25 mm cover glass (Corning) was spin-coated with a ∼25 μm layer of PDMS and the assembled device flushed with water for 24 h, which induced the migration of Pluronic F127 to the PDMS/water interface to reduce surface adhesion of sample molecules.^94^ The sample inlet of the microfluidic mixing device was filled with 20 μL of fluorescently labeled ClyA in 4 M GdmCl. Each of the two side inlets was supplied with buffer (25 mM sodium phosphate, 75 mM NaCl, 0.001% (w/v) Tween 20, 140 mM β-mercaptoethanol, pH 7). The pressures applied were 26.69 kPa (2.42 psi) to the sample inlet and 16.27 kPa (2.36 psi) to the side inlets.

### Single-Molecule FRET Data Analysis

FRET data analysis was carried out essentially as described previously^91^ with Fretica, a user-extendable Wolfram Mathematica package with a backend written in C++ for analyzing single-molecule fluorescence data (schuler.bioc.uzh.ch/programs). All measurements were recorded with pulsed interleaved excitation (PIE) to separate out bursts emitted from molecules lacking an active acceptor dye.^92^ For the construction of transfer-efficiency histograms, only photons following donor excitation were included and corrected for different quantum yields of the dyes, different detection efficiencies, crosstalk, acceptor direct excitation, and background.^95^ Only those bursts were selected that show emission after acceptor excitation, with a stoichiometry ratio (*S*) of less than 0.7. For every selected burst, the transfer efficiency was calculated according to *E* = *n*_A_/(*n*_A_ + *n*_D_), with *n*_A_ and *n*_D_ being the numbers of corrected acceptor and donor photons detected after donor excitation, respectively. The resulting transfer efficiencies were binned in a histogram.

For burst identification, contiguous photons detected after donor excitation pulses with interphoton times of less than 100 μs were combined into one fluorescence burst. In microfluidic mixing experiments, bursts were considered for data analysis if more than 40 photons were identified. For the first histogram recorded 3 ms after mixing (Figure 1d), a lower threshold of 25 photons was applied due to the higher flow velocity in the narrow initial part of the observation channel and the correspondingly reduced residence times in the confocal volume.^33^

Measurements of freely diffusing molecules in the absence of flow were analyzed with a detection threshold of 50 photons. To extract mean transfer efficiencies, the resulting histograms for the measurements in 4 M GdmCl (Figure 3a, left column) were fitted with a Gaussian peak function for symmetric peaks (variants T2C/Q56C and A187C/K290C) and a four-parameter log-normal peak function for asymmetric peaks (variants Q56C/E252C, T2C/E252C, and T2C/V303C).^54^ Measurements in the microfluidic device were all fitted with log-normal peak functions (Figure 3a, right column). Manual mixing data for refolding of the monomer were globally fitted with two Gaussian peak functions (Figure S2).

### Quantifying the Scaling Exponent from ⟨*E*⟩ of ClyA Variants in 4 M GdmCl

To take into account the presence of distance distributions in unfolded ClyA, an interdye distance distribution of a generalized version of a self-avoiding walk model was assumed.^6^ Since the excited-state lifetime of the donor is much shorter than the reconfiguration time of the chain, the mean transfer efficiencies for a given interdye distance distribution *P*(*r*) can be calculated according to^68^
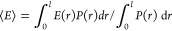
with *E*(*r*) = *R*_0_^6^/(*R*_0_^6^ + *r*^6^), where *R*_0_ is the Förster radius and *l* is the contour length of the chain. The Förster radius for the dye pair Alexa Fluor 488/Alexa Fluor 594 was calculated to be 5.6 nm at 0 M GdmCl^96^ and corrected for changes in refractive index at 4 M GdmCl. The probability density function for the self-avoiding walk model is given by^6^
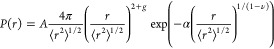
with *g* = 0.1615/ν; the constants *A* and α were obtained from the conditions ∫__0__^^∞^^*P*(*r*) d*r* = 1 and ∫__0__^^∞^^*P*(*r*) *r*^2^ d*r* = ⟨*r*^2^⟩. The scaling of the root mean squared distance ⟨*r*^2^⟩^1/2^ with segment length was calculated according to ⟨*r*^2^⟩^1/2^ = √[(2*l*_*p*_*b*)(*N* + *L*)^ν^], where *N* is the number of residues probed and *L* accounts for the contribution of both dye linkers, previously estimated to correspond to a length of nine peptide residues.^97−99^ The persistence length *l*_p_ was assumed to be 0.4 nm, and *b* is the segment length per residue (0.38 nm). The scaling exponent ν was a fit parameter, yielding ν = 0.6.

### Data Reduction by Moving Windows Analysis and Singular Value Decomposition (SVD)

For the moving window analysis, a window size of Δ*t* = 300 s was used, and histograms of overlapping time intervals were calculated (0–300, 150–450, 300–600 s, ...). The identified bursts of two independent measurements were combined for each window. For each measurement, a time *t* = *t*_d_ + *t*_s_ + Δ*t*/2 was assigned, where *t*_d_ accounts for the dead time of ∼20 s and *t*_s_ is the start time of the corresponding window. Singular value decomposition (SVD) was used as a model-free way of determining the number of components necessary to explain the changing transfer efficiency histograms during refolding of ClyA.^54^ The normalized amplitudes of each bin in a transfer efficiency histogram are written as a vector. The vectors of histograms recorded at different times after mixing are then combined into an *M* × *N* matrix **H**. This matrix can be decomposed into the product of three matrices, **H** = **USV**^T^. The orthogonal *M* × *M* matrix **U** contains information about the shape of the transfer efficiency histograms. The diagonal matrix **S** contains the singular values sorted by magnitude along the diagonal. For the data set depicted in Figure 1c, two components could be identified that clearly differ from noise (Figure 1e). The columns of the orthonormal matrix **V** contain information about the kinetics. The two significant kinetic vectors were fitted globally with a single-exponential function and a shared refolding rate coefficient (Figure 1f).

### Fluorescence Anisotropy and Lifetime Analysis

For the analysis of the steady-state donor fluorescence anisotropy, photons of the FRET population after donor excitation were selected. For the analysis of acceptor anisotropy, photons after acceptor direct excitation were chosen. As a four-channel instrument was used for data acquisition, photons could be sorted according to parallel and perpendicular polarization. The corresponding G-factors that account for different detection efficiencies for perpendicular and parallel polarized light, were determined to be 1.02 and 0.79 for donor and acceptor excitation, respectively. Additional correction factors (L factors) for the high-numerical-aperture objective were used as described previously.^34^ Anisotropies were calculated according to
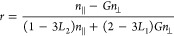
where *n*_⊥_ and *n*_∥_ denote the number of photons with perpendicular and parallel polarization relative to the excitation light, respectively (Figure S3).

To generate lifetime versus transfer efficiency plots (Figure 4), the donor fluorescence lifetime in the absence of the acceptor (τ_D_) was determined by fitting the tail of the lifetime histogram of the donor-only species (*S* > 0.9) with a single-exponential decay. The donor lifetime in the presence of the acceptor (τ_DA_) was obtained from the mean detection time of the burst photons after donor excitation.^5^

### Nanosecond Fluorescence Correlation Spectroscopy (nsFCS)

Autocorrelation curves of donor and acceptor channels as well as cross-correlation curves between donor and acceptor channels were computed as described previously.^69^ The three curves were fitted globally in the range of lag times from τ = −100 ns to τ = +100 ns with
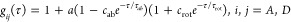
where the amplitude *a* depends on the mean number of molecules in the confocal volume and the background signal. *c*_ab_ and *c*_rot_ are the amplitudes related to photon antibunching and rotational dynamics, respectively. The corresponding relaxation times are τ_ab_ and τ_rot_, which are global fit parameters, whereas *a*, *c*_ab_, and *c*_rot_ were fitted individually for each correlation curve (Figure 4).

### Computer Simulations

Plain MD simulations in explicit solvent were performed using the CHARMM36 force field^73^ and Amber 14SB^74^ in TIP3P water. Implicit solvent plain MD simulations employed the Amber 14SB force field using the solvation model implemented in GROMACS 4.6.5.^100^ In this approach, the Born radii are calculated according to the Onufriev–Bashford–Case algorithm.^101^ The hydrophobic propensity of nonpolar residues is taken into account through an interaction term proportional to the atomic solvent accessible surface area. The solvent-exposed surface of the different atoms is calculated from the Born radii, according to the approximation developed by Schaefer, Bartels, and Karplus.^102^ All biased simulations where performed using the Amber 14SB force field in explicit TIP3P water. In all simulations, we adopted a stochastic velocity rescaling thermostat,^103^ with bond lengths held fixed and an integration time step of 2 fs. We did not explicitly include the fluorophores in the protein structure. The internal mobility of the chain in the collapsed intermediate was assessed by computing the residue-wise root-mean-square fluctuations. The results for 15 implicit-solvent (Amber 14SB) and four explicit-solvent simulations (CHARMM36), after averaging over the residues yielded 0.62 ± 0.11 nm and 0.64 ± 0.17 nm, respectively. The comparable fluctuations suggest that the implicit-solvent simulations provide a reasonable description of the system.

In rMD simulations, the equations of motion for all atoms in the protein are modified by adding the biasing force *F*^*i*^_*rMD*_(*X, t*), defined as follows
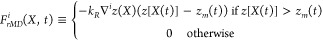
where *z*(*X*) is a CV that measures the overlap between the instantaneous and the native contact map:
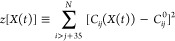
where
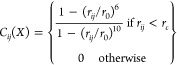
and *C*_*ij*_^0^ is the same function evaluated for the native crystal structure (target). *r*_0_*=* 0.75 nm is a conventional reference distance for contact formation, and *r*_*c*_ = 1.23 nm is a cutoff distance that is employed to increase the computational efficiency of the algorithm by restricting the calculation to spatially close atoms. The variable *z*_*m*_(*t*) in the definition of the biasing force denotes the smallest value attained by the variable *z*(*X*(*t*)) up to time *t*. The coupling constant *k*_*R*_ defining the strength of the biasing force was set to 0.00025 kJ/mol. With this choice, the modulus of the biasing force was always at least 2 orders of magnitude smaller than the modulus of the total physical force. We rescale the CV *z*(*X*) to vanish in fully denatured configurations and to reach unity in the target configuration. Such a rescaled CV represents our proxy of the committor function.

To implement the BF approach, we first randomly selected 10 configurations in the folding intermediate. Then, from each of such configurations, we computed 20 rMD trial folding trajectories, each lasting for 1.5 nominal nanoseconds. The trial trajectories generated from each initial condition were ranked by computing the bias functional
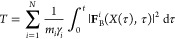
In this equation, γ_i_ and *m*_i_ are, respectively, the viscosity and mass of the *i*th particle, and |**F**_B_^*i*^(*X*(τ),τ)|^2^ is the square modulus of the biasing force, integrated for all times along the rMD trajectory, *X*(τ). It can be shown that the rMD trial trajectories with the lowest value of this functional are those with the highest probability to occur in completely unbiased simulations. In this sense, such least biased trajectories provide a variational estimate of the folding pathways generated by the Langevin dynamics. The BF scheme has been extensively validated using plain MD protein folding simulations performed on the Anton supercomputer,^81^ and against the results of deuterium-exchange experiments^104^ and time-resolved near-UV CD measurements.^105^ Its high computational efficiency enables tens of folding trajectories for proteins containing even several hundred amino acids and folding times in the minutes range to be generated on a standard CPU cluster or a small GPU server.^106^

To identify kinetically relevant folding intermediates, we projected calculated trajectories generated by MD or rMD on the plane selected by the fraction of native contacts *Q* and the instantaneous radius of gyration, *R*_g_ (Figure 5). Metastable states were identified as peaks in this density plot. In BF calculations, only configurations extracted from the least biased trajectories were used to compute observables (Figure 5). This method to identify folding intermediates has been successfully applied in a number of previous works with results in good agreement with experiments.^104,105^ FRET efficiencies of (meta-)stable states were computed from an ensemble average of the efficiencies of microscopic protein configurations using the point dipole approximation and assuming rapid orientational averaging of the fluorophores.

## Supporting Information Available

Expected and measured transfer efficiencies, ClyA refolding kinetics, fluorescence anisotropies, nanosecond dynamics, chain collapse from simulations, comparison of collective variables (PDF)

## Abbreviations

ClyA: cytolysin A
FRET: Förster resonance energy transfer
CD: circular dichroism
MD: molecular dynamics
rMD: ratchet-and-pawl molecular dynamics
BF: bias functional
PDMS: polydimethylsiloxane
GdmCl: guanidinium chloride
SVD: singular value decomposition
DTT: dithiothreitol
DMSO: Dimethyl sulfoxide
IMAC: immobilized metal affinity chromatography
NTA: nitrilotriacetic acid
PIE: pulsed interleaved excitation
nsFCS: nanosecond fluorescence correlation spectroscopy
