# Supplementary material for: Slow Escape from a Helical Misfolded State of the Pore-Forming Toxin Cytolysin A
Source: JACS Au. 2021 Jul 13;1(8):1217–30. doi: 10.1021/jacsau.1c00175 (PMC8397351; doi:10.1021/jacsau.1c00175)
Supplement: Supplementary file 1 — au1c00175_si_001.pdf [file au1c00175_si_001.pdf]

Supporting Information for

Slow escape from a helical misfolded state of the pore-forming toxin Cytolysin A

Fabian Dingfelder[1], Iuri Macocco[2 3], Stephan Benke[1], Daniel Nettels[1], Pietro Faccioli[2 4] & Benjamin Schuler[1,5]

[1]Department of Biochemistry, University of Zurich, Winterthurerstrasse 190, 8057 Zurich, Switzerland
[2]Department of Physics, Trento University, Via Sommarive 14, 38123 Povo (Trento), Italy
[3] SISSA, Via Bonomea 265, 34136 Trieste, Italy.
[4] INFN-TIFPA, Via Sommarive 14, 38123 Povo (Trento), Italy
[5]Department of Physics, University of Zurich, Winterthurerstrasse 190, 8057 Zurich, Switzerland

Email: pietro.faccioli@unitn.it, schuler@bioc.uzh.ch

a

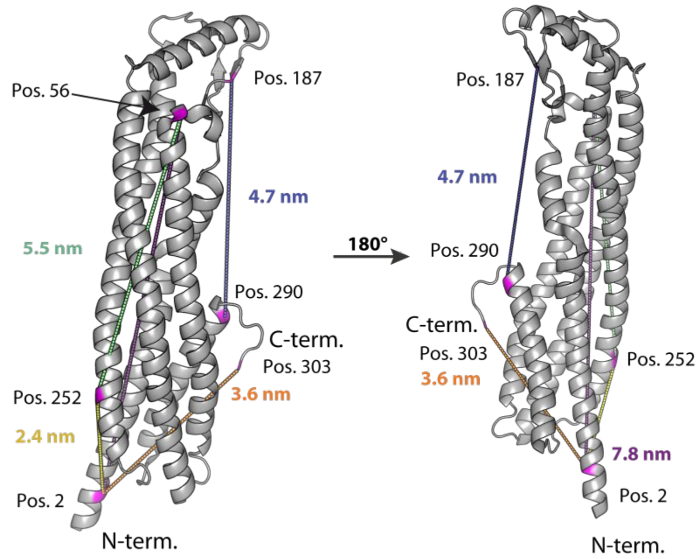

b

Variant	<E$_{calc.}$>	<E$_{meas.}$>
2/56	$0.075^{+4\cdot10^{-4}}_{-0.002}$	0.08 ± 0.03
187/290	$0.83^{+4\cdot10^{-4}}_{-0.002}$	0.83 ± 0.03
56/252	$0.40^{+0.02}_{-0.04}$	0.35 ± 0.03
2/252	$0.91^{+0.01}_{-0.02}$	0.87 ± 0.03
2/303	$0.69^{+0.03}_{-0.05}$	0.55 ± 0.03

Figure S1: Expected and measured transfer efficiencies. (a) Cartoon representation of the crystal structure of the ClyA monomer (PBD code 1QOY)[1]. The residues used for labelling are highlighted in magenta. The distances between the corresponding Cα-atoms of the different variants investigated are displayed as colored lines, following the same color code as in Figure 1 and Figure S2. The last five C-terminal residues are not resolved in the crystal structure. Thus, residue 298 is highlighted instead of residue 303 that was used for labeling. (b) Mean transfer efficiencies of the five different variants estimated based on the crystal structure via the accessible volumes of the dyes[2] and their translational dynamics[3] and measured experimentally in the microfluidic mixing device after ~1 min. Note that variant 187/290 did not refold in the microfluidic device. We thus report the mean transfer efficiency obtained in the manual mixing experiment (Figure S2) for this variant.

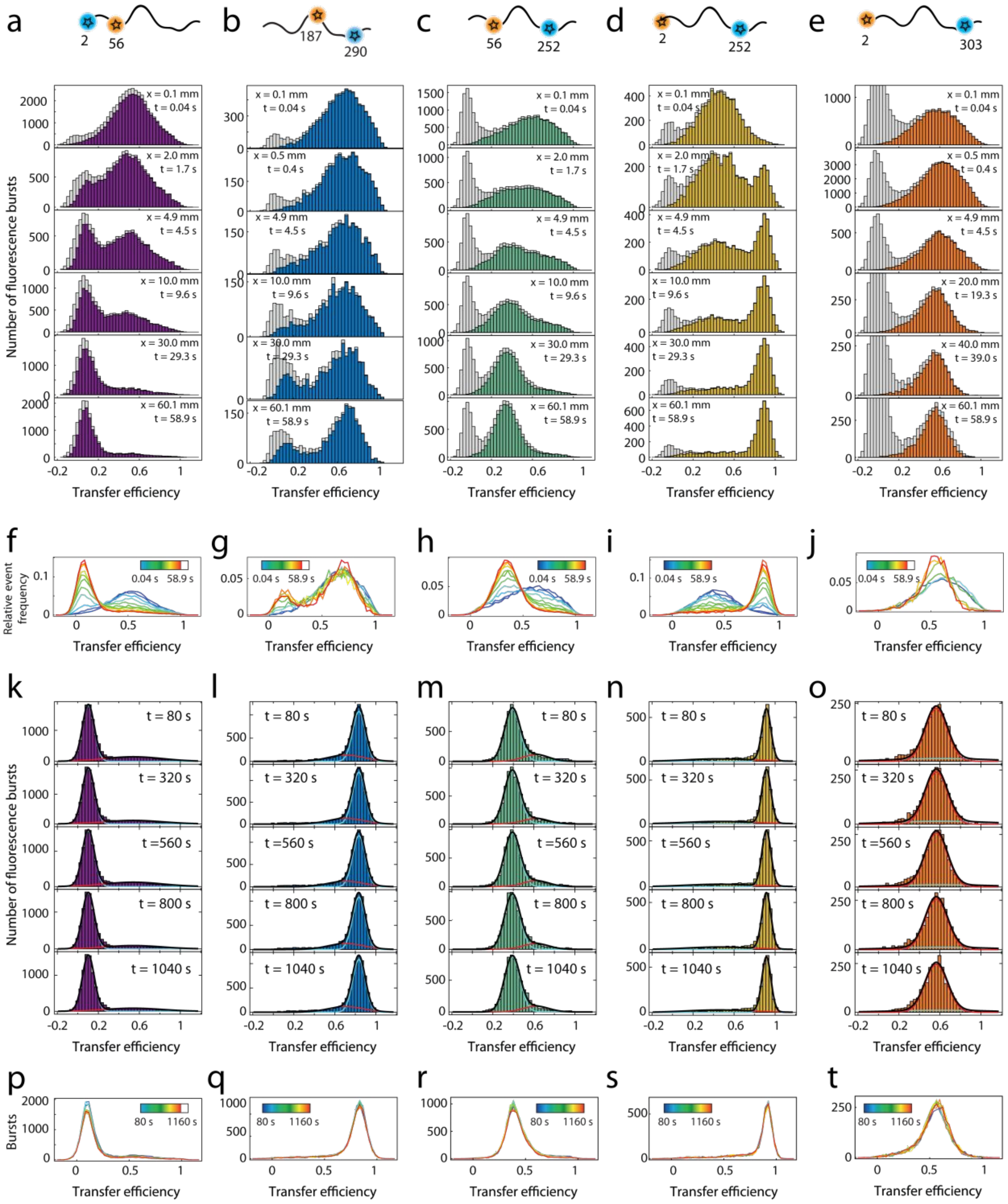

Figure S2: Refolding of ClyA studied by microfluidic and manual mixing in combination with single-molecule FRET. Folding was triggered by rapid dilution from 4 M GdmCl to 0.4 M GdmCl. (a-e) Time series of transfer efficiency histograms of the different ClyA variants recorded in the microfluidic device covering times after mixing up to ~1 min. Positions and times after mixing are indicated in the corresponding transfer efficiency histograms. Gray histograms are from all identified bursts, histograms in color are from bursts originating from the FRET-population isolated by pulsed interleaved excitation (stoichiometry > 0.7, see Materials and Methods for details). The histograms shown in (d) correspond to the data shown in Figure 1. One of the five variants investigated (A187C/K290C) did not refold in the microfluidic device. (f-j) Overlay of the transfer efficiency histograms for the respective variants recorded after microfluidic mixing. (k-o) Time series of transfer efficiency histograms of the different ClyA variants recorded after manual mixing (dead time ~20 s). Times after mixing are indicated in the transfer efficiency histograms. The time series of each variant was fitted globally with two Gaussian peak functions, and the position of the intermediate state was constrained to the mean transfer efficiencies determined in the microfluidic mixing device. (p-t) Overlay of all transfer efficiency histograms for the respective variants recorded after manual mixing, indicating only minor changes as a function of time.

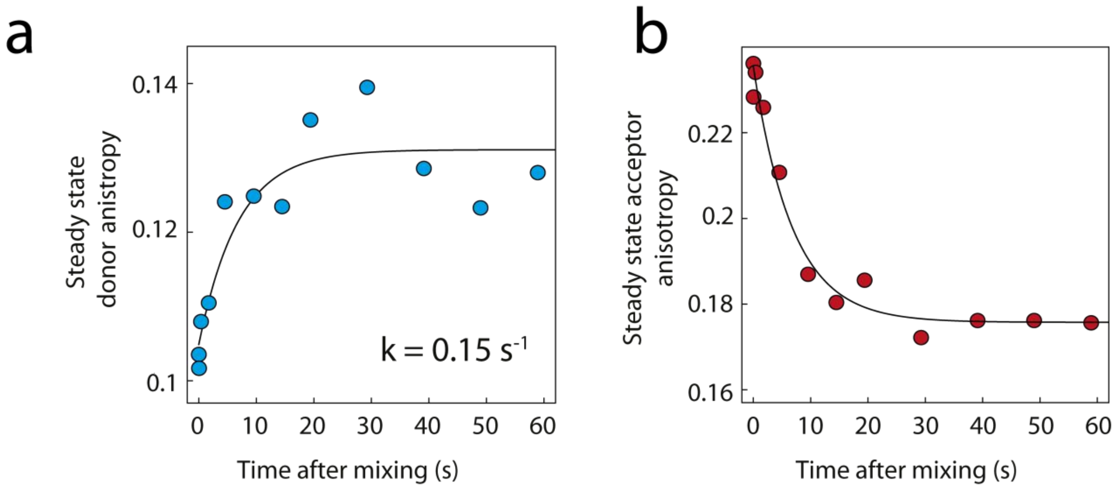

Figure S3: Determination of steady-state fluorescence anisotropies. (a, b) Steady-state donor and acceptor anisotropies after acceptor excitation as a function of time after mixing for ClyA 2/252 measured in the microfluidic device (see Figure 1c and Figure S2). Anisotropies do not change much during refolding, and especially the donor anisotropies are low throughout. Thus, slow orientational relaxation of the fluorescent dyes is unlikely to dominate the broadening of the transfer efficiency histograms beyond shot-noise (Figure 1, Figure S2). The solid black line shows a global single-exponential fit with a shared rate coefficient *k*.

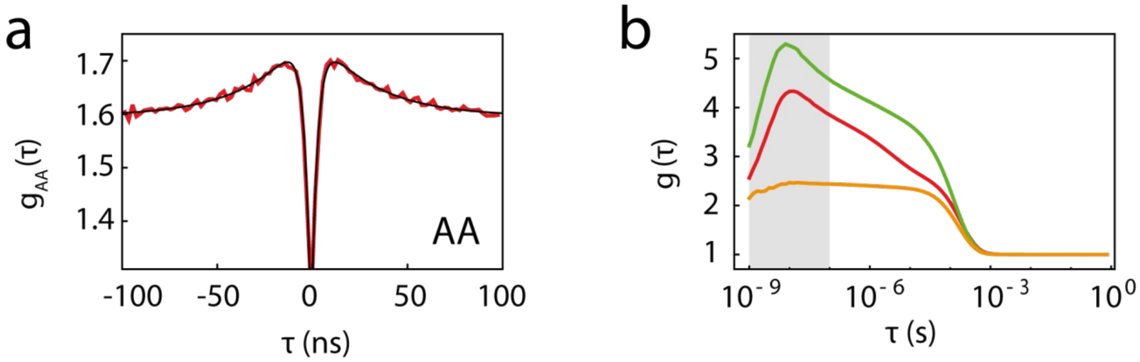

Figure S4: Investigating nanosecond dynamics with fluorescence correlation spectroscopy. (a) Nanosecond fluorescence correlation spectroscopy (nsFCS) measurement of the intermediate state of ClyA 2/252 recorded in the microfluidic device. The panel shows the acceptor-acceptor autocorrelation upon direct excitation of the acceptor, demonstrating that the positive correlation observed is not caused by distance dynamics. Similar to the data shown in Figure 4, a two-component fit was applied, yielding $\tau_{ab} = 3.3$ ns and $\tau_{rot} = 30$ ns. (b) Autocorrelation of acceptor fluorescence (red), donor fluorescence (green) and cross correlation of donor and acceptor fluorescence (orange) displayed on a logarithmic timescale from 1 ns to 1 s.

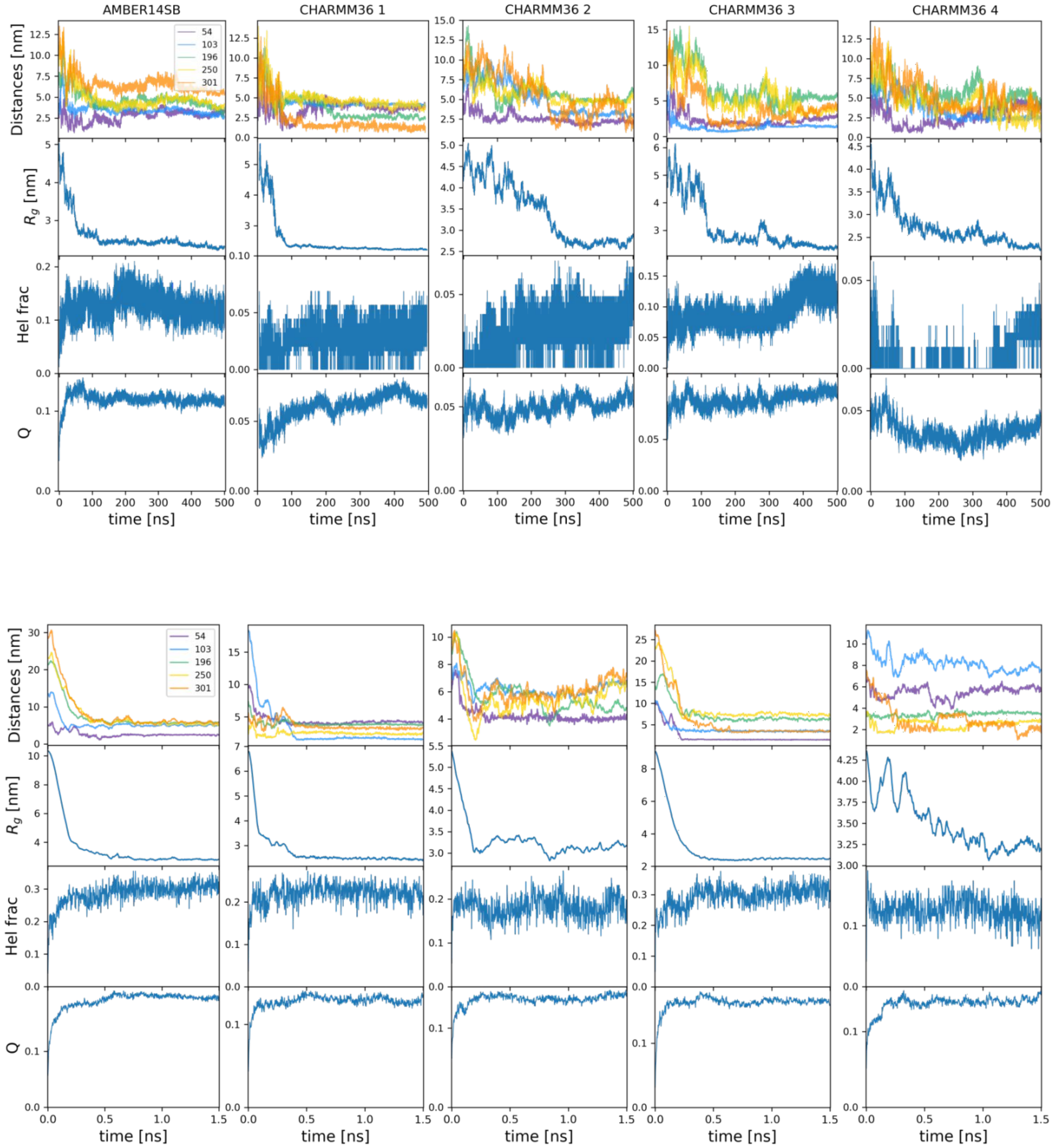

Figure S5: Chain collapse to the compact intermediate in MD simulations. Time evolution of different observables calculated from different MD simulations starting from fully unfolded configurations. From top to bottom: distances between the residue pairs monitored by FRET experiments (legend indicates sequence separation of the FRET labeling variants), radius of gyration, R_g, helicity relative to the native state in the crystal structure (Hel frac), and the fraction of native contacts, Q. The upper panels report the results of simulations in explicit solvent (with Amber 14SB and CHARMM36 force fields as indicated). The lower panels report examples from simulations using Amber 14SB in implicit solvent.

**Unfolded State**	R_g [nm]	Q	Hel. Fract.
CHARMM36	5.1 ± 0.9	0.04 ± 0.01	0.01 ± 0.01
Amber14SB (implicit solvent)	8.1 ± 2.5	0.03 ± 0.02	0.05 ± 0.02
**Molten Globule State**			
CHARMM36	2.50 ± 0.25	0.07 ± 0.02	0.06 ± 0.04
Amber14SB (explicit solvent)	2.45 ± 0.17	0.11 ± 0.01	0.14 ± 0.02
Amber14SB (implicit solvent)	3.00 ± 0.45	0.15 ± 0.02	0.21 ± 0.07
**Native State**			
CHARMM36	2.88 ± 0.02	0.98 ± 0.01	0.98 ± 0.02
Amber 14SB (implicit solvent)	2.84 ± 0.02	0.93 ± 0.01	0.99 ± 0.01

Table S1: Comparison of collective variables in the unfolded, molten globule and native state in different simulation models. Radius of gyration and fraction of native contacts in the molten globule state are similar in the different force fields. The helicity is reported relative to the value in the energy-minimized native structure and was computed using the DSSP algorithm[4]. Observables in the native state were calculated from short MD simulations started from the energy-minimized crystal structure[1].

SI References

1. Wallace, A. J.; Stillman, T. J.; Atkins, A.; Jamieson, S. J.; Bullough, P. A.; Green, J.; Artymiuk, P. J., E. coli Hemolysin E (HlyE, ClyA, SheA). *Cell* **2000,** *100* (2), 265-276.
2. Kalinin, S.; Peulen, T.; Sindbert, S.; Rothwell, P. J.; Berger, S.; Restle, T.; Goody, R. S.; Gohlke, H.; Seidel, C. A. M., A toolkit and benchmark study for FRET-restrained high-precision structural modeling. *Nature Methods* **2012,** *9* (12), 1218-1225.
3. Dingfelder, F.; Benke, S.; Nettels, D.; Schuler, B., Mapping an Equilibrium Folding Intermediate of the Cytolytic Pore Toxin ClyA with Single-Molecule FRET. *The Journal of Physical Chemistry B* **2018,** *122* (49), 11251-11261.
4. Kabsch, W.; Sander, C., Dictionary of protein secondary structure: pattern recognition of hydrogen-bonded and geometrical features. *Biopolymers* **1983,** *22* (12), 2577-637 issn: 0006-3525.
